# Multi-Level and Multi-Scale Feature Aggregation Network for Semantic Segmentation in Vehicle-Mounted Scenes

**DOI:** 10.3390/s21093270

**Published:** 2021-05-09

**Authors:** Yong Liao, Qiong Liu

**Affiliations:** School of Software Engineering, South China University of Technology, Guangzhou 510006, China; 201821038512@mail.scut.edu.cn

**Keywords:** multi-scale feature extraction, feature aggregation, real-time semantic segmentation, vehicle-mounted scenes

## Abstract

The main challenges of semantic segmentation in vehicle-mounted scenes are object scale variation and trading off model accuracy and efficiency. Lightweight backbone networks for semantic segmentation usually extract single-scale features layer-by-layer only by using a fixed receptive field. Most modern real-time semantic segmentation networks heavily compromise spatial details when encoding semantics, and sacrifice accuracy for speed. Many improving strategies adopt dilated convolution and add a sub-network, in which either intensive computation or redundant parameters are brought. We propose a multi-level and multi-scale feature aggregation network (MMFANet). A spatial pyramid module is designed by cascading dilated convolutions with different receptive fields to extract multi-scale features layer-by-layer. Subseqently, a lightweight backbone network is built by reducing the feature channel capacity of the module. To improve the accuracy of our network, we design two additional modules to separately capture spatial details and high-level semantics from the backbone network without significantly increasing the computation cost. Comprehensive experimental results show that our model achieves 79.3% MIoU on the Cityscapes test dataset at a speed of 58.5 FPS, and it is more accurate than SwiftNet (75.5% MIoU). Furthermore, the number of parameters of our model is at least 53.38% less than that of other state-of-the-art models.

## 1. Introduction

**Figure 1:**
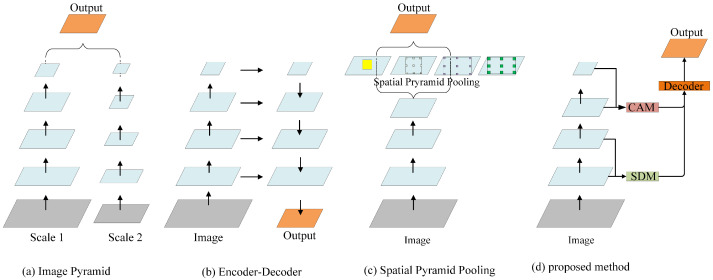
Illustrating network structures for multi-scale feature representations. CAM: context aggregation module. SDM: spatial detail module.

Semantic segmentation is a basic computer vision topic, wherein an explicit category label is assigned to each pixel of an input image, which can be utilized in many applications, such as automotive driving, medical imaging, and video surveillance [1,2,3,4]. In vehicle-mounted scenes, objects usually appear at multiple scales, because there are various objects, and varying locations or distances of these objects; this is one of the great challenges in semantic segmentation. In general, convolutional neural networks (CNNs) are inherently limited by the design of the neurons at each layer, where the receptive field is restricted to constant regions, and the representation ability of multi-scale features is limited. Extensive efforts [5,6,7,8] have been made to acquire multi-scale feature representation. The pioneering work of multi-scale feature representation involves constructing an image pyramid (Figure 1a), where small object details and long-range context can be obtained from large-scale and small-scale inputs, respectively [7,9,10]. However, this process takes a lot of time. In order to mitigate this problem, other researchers extract features hierarchically by an encoder–decoder structure (Figure 1b) [11,12,13]. Because there are different neuron receptive fields in different layers, the features extracted from various layers of an encoder implicitly contain different scale information. However, each layer of an encoder transmits the same feature maps to the corresponding layer of a decoder, which results in double calculation. A more efficient method builds a spatial pyramid feature extraction module (Figure 1c), such as a pyramid pooling module [8] or atrous spatial pyramid pooling (ASPP) module [5]. Leveraging one of them enables the output features of a large-scale backbone network to be passed to different receptive field branches and transformed into multi-scale features. Furthermore, an enhanced feature representation is obtained by merging all branch features. Nevertheless, these approaches usually apply complicated backbone networks (such as VGG [14] or ResNet-101 [15]). Therefore, their computational overhead is huge.

**Figure 2:**
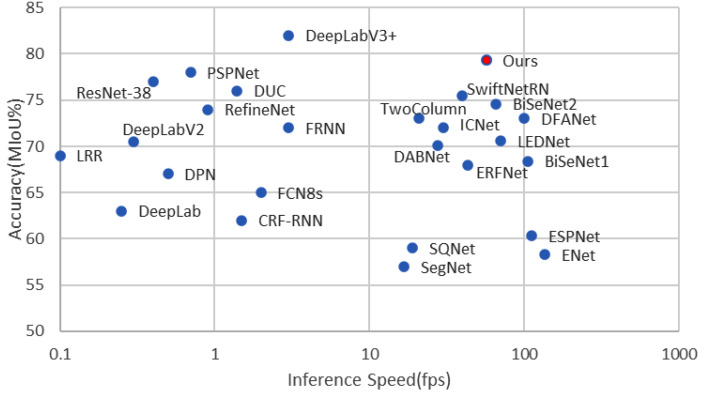
The accuracy (mIoU) and inference speed (fps) obtained by several state-of-the-art methods on the Cityscapes test dataset.

A semantic segmentation network used in a vehicle-mounted scene not only needs enough accuracy, but it must be able to operate in real time. In the past decade, many model compression methods have been proposed, such as network pruning [16], singular value decomposition (SVD) [17], and low-rank factorization [18]. However, these methods are generally used in well-trained, complicated backbone networks, and they are rarely applied to lightweight networks. Some real-time semantic segmentation methods build a network more suitable for mobile devices by using a lightweight backbone network that is composed of deep separable convolution [19,20,21,22,23,24,25]. They significantly reduce network parameters by compressing feature channels and reducing layers. However, parameter reduction is usually accompanied by decreasing segmentation accuracy. The final mean intersection over union (MIoU) score of these models notably drops to 71% or even lower, which limits their practical application. In contrast, other methods use ResNet-18 [15] as a backbone network to obtain high accuracy [26,27,28,29,30,31], but they also have relatively more parameters and computations. It is a great challenge to strike a good trade-off between model accuracy and efficiency. To illustrate this actuality, Figure 2 presents the accuracy (mIoU) and inference speed (frames per second (fps)) obtained by several state-of-the art methods and our proposed method on the Cityscapes test dataset.

Many state-of-the-art semantic segmentation methods illustrate that spatial details and high-level semantic context information are the keys to obtaining high accuracy [27,32]. With the deepening of a network, the resolution of the feature map will decrease, and more spatial details will be lost. Dilated convolution is a general technique for preserving spatial details, which generates high-resolution feature maps without increasing parameters. Some state-of-the-art methods apply dilated convolutions at the last several stages of their networks to construct the dilated FCN [5,33]. However, the dilated FCN makes the network wider and computationally intensive. When compared with the original FCN [34], 23 residual blocks in the dilated FCN (based on ResNet-101 [15]) require four times more computational resources and memory, while three residual blocks take 16 times more resources [35]. This is clearly not suitable for real-time semantic segmentation networks. Designing additional shallow and wide sub-networks to extract spatial details is a simple and effective solution to this. BiSeNet [27] and ICNet [30] are representative approaches. They extend an image pyramid structure to a multi-branch structure, and extract spatial details and context semantics in parallel. Nevertheless, an additional sub-network not only brings redundant parameters, but it also slows down the network to a certain extent. Generally speaking, semantic contextual information is more concentrated in the higher levels of a network. For this reason, many context extraction modules, such as ASPP [5], PPM [8], and attention module [33], have been proposed to connect to the tail of a network. However, some studies have shown that their effectiveness depends on complicated backbone networks with a large channel capacity [19].

In this paper, we propose a multi-layer and multi-scale feature aggregation network (MMFANet) for semantic segmentation in vehicle-mounted scenes. Different from previous approaches that only focus on the accuracy or efficiency of the model, we aim to improve model efficiency while simultaneously ensuring high segmentation accuracy on multi-scale objects. In pursuit of better accuracy, we adopt the following design. Firstly, a cascaded dilated convolution module (CDCM) is designed to enable the backbone network to perform multi-scale feature extraction layer-by-layer. Subsequently, a context aggregation module (CAM), which is composed of a channel attention mechanism and CDCM, can adaptively encode the multi-scale context information and channel context information under a larger receptive field. Thus, the CAM guides the learning process more precisely. Next, a spatial detail module (SDM) is designed to ease the transmitting of low-frequency information from low layers to high layers. The SDM only contains two standard convolutions, and it does not require an additional sub-network or adopt a dilated FCN to generate high-resolution features. Finally, a decoder is adopted to jointly learn spatial detail and multi-scale context information, and generates final segmentation results. In order to improve the model efficiency, we optimized ResNet-18 [15]. We use a CDCM with lower feature channel capacity to replace the residual blocks of the last two stages of ResNet-18 (ResNet is divided into five stages according to the resolution of output feature maps). The maximum feature channel number of our backbone network in each layer is no more than 256, which maintains the computation efficiency and simultaneously guarantees adequate information during propagation. The modified lightweight backbone ResNet-18 is named MSResNet-18 in this paper. The lower channel capacity of MSResNet-18 avoids a large number of channel calculations. Figure 1d illustrates the proposed network architecture. Without any ImageNet pretraining, MMFANet obtains 79.3% MIoU on the Cityscapes test set, and 68.1% MIoU on the CamVid test set, with only 5.5 M parameters. Meanwhile, it processes an image of 1536 × 768 resolutions at a speed of 58.5 FPS on a single NVIDIA GTX 1080Ti card. the experimental results demonstrate that our method achieves a better trade-off between model accuracy and computational complexity. The contributions of this paper are listed below:

1. A cascade dilated convolution module (CDCM) is proposed, and it is used as a building block of the backbone network to efficiently extract multi-scale features layer by layer. By controlling the channel capacity of the module, we build a more efficient and lightweight backbone network, i.e., MSResNet-18.
2. A lightweight feature aggregation framework (consisting of an SDM and CAM) is proposed to fuse the features of each stage of our backbone network. Therefore, it can asymptotically refine segmentation results in a coarse level for better accuracy.
3. The design of MMFANet guarantees high segmentation accuracy while significantly improving network efficiency. When compared with state-of-the-art methods, MMFANet can obtain a better trade-off between model accuracy and efficiency. We release our source code for the benefit of other researchers and further advancement in this field. The Pytorch source code used for this research is available at GitHub: https://github.com/GitHubLiaoYong/MMFANet (accessed on 1 July 2020).

## 2. Related Works

In recent years, deep convolutional neural network (DCNN)-based semantic segmentation methods have made great progress. In the following subsections, we will review the DCNN-based semantic segmentation methods that are most relevant to our work. We will introduce both multi-scale object segmentation methods and real-time semantic segmentation methods. In addition, we will review several DCNN methods that focus on integrating spatial details and context information.

### 2.1. Multi-Scale Object Segmentation

Multi-scale feature representation is very important to the segmentation of multi-scale objects [5]. Only using single-scale features is often suboptimal [34]. Farabet et al. [9] input multi-scale images that are generated by a Laplacian pyramid into a group of convolutional networks that share weights, and use channel concatenation to fuse all the network features. Eigen et al. [10] progressively refines predictions using a sequence of scales, and captures many image details without any superpixels or low-level segmentation. Based on the image pyramid, Chen et al. [7] further propose an attention mechanism that learns to softly weigh the multi-scale features at each pixel location. The main disadvantage of this type of method is that processing multi-scale images is not only time-consuming, but also requires more GPU memory. Features within a network are multi-scale in nature, as neurons in different layers of a network have different receptive fields. Therefore, studies [11,12] introduce an encoder–decoder structure (Figure 1b). U-Net [11] adopts a contracting path to capture context and a symmetric expanding path to fuse the hierarchical features of the backbone. Analogously, SegNet [12] adopts a typical U-Shape structure and utilizes the saved pooling indices that are computed in the max-pooling step of the encoder to gradually incorporate different resolution features. This kind of method is similar to the FPN [36] structure in object detection, which improves the performance by reusing features of various resolutions. Nevertheless, this symmetrical encoder–decoder structure brings double computing overhead. A relatively more effective method uses a spatial pyramid structure (Figure 1c). PSPNet [8] employs a pyramid pooling module (PPM) to aggregate contextual information from multi-scale regions. Moreover, an atrous spatial pyramid pooling (ASPP) module is developed in DeepLabv3 [6], which has multiple paths of different dilated convolutions to extract multi-scale context information. Different from the above methods, we embed a multi-scale feature extraction module into the backbone network, and design an effective feature aggregation framework (consisting of an SDM and CAM) to aggregate multi-level features.

### 2.2. Real-Time Semantic Segmentation

Current real-time semantic segmentation models can be generally divided into two streams. The methods in the first category employ existing lightweight backbone networks (like MobileNet [37] and ShuffleNet [38]) for acceleration. ICNet [30] lets low-resolution images go through the full semantic perception network to generate a coarse prediction map, and then adopts a cascade feature fusion unit to integrate medium- and high-resolution features. BiSeNet [27] designs a spatial path and a semantic path: the spatial path extracts spatial information, while the semantic path extracts contextual information. SwiftNet [26] seeks a simpler U-shaped structure to reuse the multi-scale features in the backbone network, and leverages lightweight upsampling with lateral connections as the most cost-effective solution to restore the prediction resolution. Meanwhile, DFANet [31] uses a deep feature aggregation structure aggregating discriminative features through a sub-network and sub-stage cascade. The other type of real-time semantic segmentation model uses a custom lightweight architecture, and it compresses the width and depth of the network to construct a real-time semantic segmentation network. For example, ERFNet [23] combines the residual structure with convolution decomposition to reduce computational overhead while ensuring accuracy. ESPNet [24] and ESPNetv2 [25] both utilize an efficient spatial pyramid (ESP) module, where standard convolution is decomposed into point-wise convolution and dilated spatial pyramid convolution. DABNet [19] adopts convolution decomposition and dilated convolution to design deeply asymmetric convolution modules, which are used as building blocks. In the present paper, we combine the advantages of both categories of real-time semantic segmentation models. More specifically, we optimize the existing lightweight backbone ResNet-18 [15] with a custom lightweight architecture.

### 2.3. Spatial Details and Semantic Context Information

Semantic segmentation not only needs to classify each pixel, but it also needs to locate them. The deep convolutional neural network (DCNN) learns very abstract feature representations by repeatedly stacking convolutional layers and subsampling layers. Deeper stages contain more semantic information (or context information), but lose too many spatial details due to subsampling layers. Conversely, shallower layers contain more spatial details, but also much irrelevant noise. The reasonable integration of these two types of features has the potential to improve the performance of semantic segmentation. FCN8s [34] fuse features from intermediate layers to compensate for spatial information of high-level features, which improves MIoU accuracy by approximately 3%. RefineNet [13] refines low-resolution (coarse) semantic features with fine-grained low-level features in a recursive manner to generate high-resolution semantic feature maps. DeepLabV3+ [32] adopts a spatial pyramid pooling module to encode multi-scale contextual information while gradually recovering spatial information through an encode–decoder structure. Li et al. [39] propose a flow alignment module to learn the semantic flow between feature maps of adjacent levels. They extended the module to a common feature pyramid structure to attain high resolution feature maps with strong semantic representation. In the present paper, we design two lightweight modules to extract spatial detail information and semantic information from different levels of the backbone network.

## 3. Proposed Method

### 3.1. Overview

**Figure 3:**
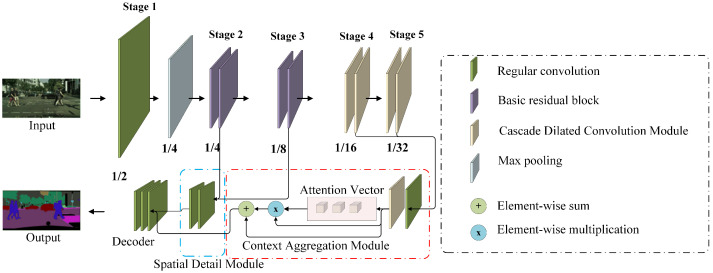
An overview of the multi-level and multi-scale feature aggregation network. Among them, 1/n represents that the resolution of the feature of this stage is 1/n of the original image resolution (n = 2, 4, 8, 16, 32).

In this section, we introduce a multi-layer and multi-scale feature aggregation network (MMFANet), which consists of four key components: a modified ResNet-18 [15] that is based on a cascade dilated convolution module (CDCM), a spatial detail module (SDM), a context aggregation module (CAM), and a decoder. MMFANet aims to reduce model complexity while achieving high accuracy by efficiently learning spatial details and context information. We first feed a given input into our modified backbone network to obtain multi-scale features, as depicted in Figure 3. More specifically, we retain the initial convolution layer, max pooling layer, and four residual blocks in the low layers of ResNet-18, but replace the residual blocks in the last two stages with the CDCM. By reducing the channel capacity of the CDCM, the number of parameters of the origin ResNet-18 is reduced by more than 74%. Next, we construct a spatial detail module (SDM), as shown in the blue box of Figure 3. Its inputs come from the output of the first two stages of the backbone network with resolutions of 1/4 and 1/8 of the input image. Thereafter, we feed multi-scale semantic features that are outputted from the last two stages of the backbone into the context aggregation module (CAM) to gather both spatial and global context information, as shown in the red box of Figure 3. We adopt the CDCM to capture multi-scale context at the spatial level according to the object scales, and the dilation rates of the CDCM are set as D={1,5,7,19} to maintain the field of view. Moreover, we use a global pooling operation to generate a group of attention vectors to capture global context at the channel level. Finally, the modified decoder in DeepLabV3+ [32] is connected to the tail of the SDM and CAM to obtain the final prediction. In the following sections, we cover the details of the CDCM, SDM, and CAM. We also summarize all of the notations in Table 1.

### 3.2. Cascade Dilated Convolution Module (CDCM)

In the task of semantic segmentation, most modern methods design multi-scale modules, such as the PPM [8] and ASPP [5], to obtain enough receptive fields while capturing objects as well as context at multiple scales. However, directly extending these methods into a real-time semantic segmentation network usually yields limited performance improvements and brings more computational cost, mainly because these modules require input feature maps that have a large number of feature channels (such as 2048). In general, a deeper network can capture a larger receptive field and encode more abstract semantic context. For example, the residual structure shown in [15] is designed to deepen a network to 1000 layers, which significantly improves the recognition ability. However, most existing lightweight backbone networks reduce the computational cost by reducing network layers, which limits the effective receptive field.

**Figure 4:**
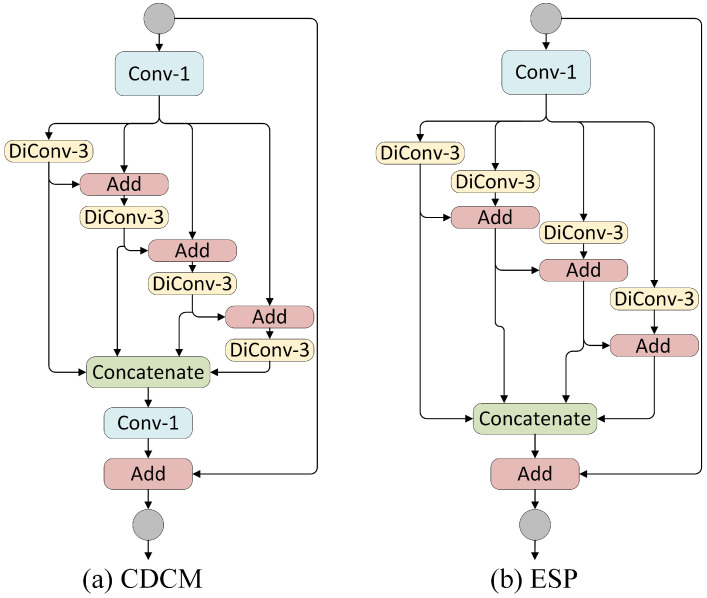
Network architecture of the ESP module and CDCM. Conv-1: 1×1 standard convolution, DiConv-n: n×n dilated convolution. The circles represent input and output feature maps.

In order to solve the above problems, and inspired by the efficient spatial pyramid (ESP) module [24] and Res2Net [36], we designed a CDCM to capture enough receptive fields while extracting multi-scale features in a more efficient way. Unlike the multi-scale modules that are connected to the tail of a backbone network, our CDCM is used as a building block. Figure 4 shows the difference between the ESP module and CDCM. In the ESP module, all of the dilated convolution branches are calculated in parallel. The branch with the largest dilated rate determines its receptive field. Thereafter, all of the neurons in each branch share the same field of view on a single scale, which makes it difficult to deal with scale variation cases in semantic segmentation. In the CDCM, the output of a dilated convolution branch is used as the input of the next branch. This can be expressed mathematically by Equation (1):

(1) $$y_{i}=\left\{\begin{array}{ccc} k_{i}\left(x\right),i & = & 1\\ k_{i}\left(x+y_{i-1}\right),i & > & 1 \end{array},\right.$$

where $k_{i}$, *x*, and $y_{i}$ are the convolution operation, input features, and output features of the *i*-th branch, respectively.

**Figure 5:**
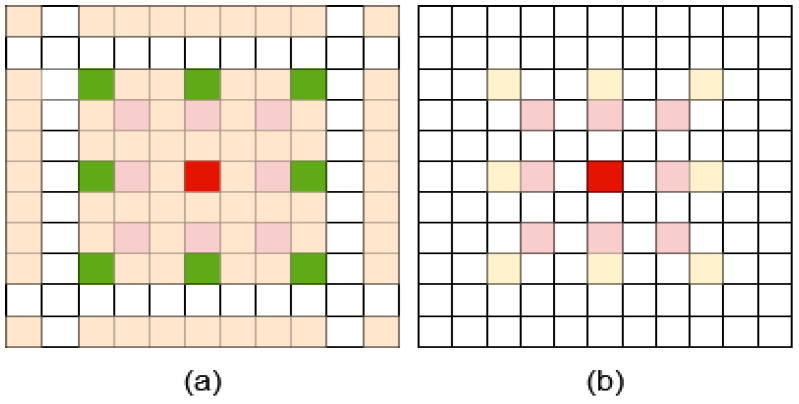
Different pixel utilization of two modes for 3×3 dilated convolution. Here, we use two dilation convolutions whose dilation ratios are 2 and 3. (**a**) Given the center point (red color), the cascade calculation mode makes 81 pixels contribute to the value of the center point. (**b**) In the parallel calculation mode, only the values of 17 pixels contribute to the final value of the center point (red color).

There are two benefits of using a cascade calculation. Firstly, the sparse sampling method of dilated convolution makes fewer feature points in a feature map participate in the calculation, which makes it impossible to fully utilize the information in the given feature map. Meanwhile, cascade calculation makes more pixels involved in the computation of feature maps. Figure 5 illustrates the different pixel utilization of two modes. Secondly, a larger receptive field can be obtained. The receptive field of the i-th branch of the CDCM can be calculated by Equation (2).

(2) $$Rec_{i}=Rec_{i-1}+2*r_{i}$$

Here, $Rec_{i}$ represents the receptive field of the i-th branch, $r_{i}$ represents the dilated rate of the i-th branch (our dilated rate setting follows the hybrid dilated convolution (HDC) mode [40], which can better overcome the gridding artifacts). More specifically, when the ESP module and CDCM both contain four branches and apply a group of dilation rates D={1,2,4,8}, the receptive field of the CDCM reaches 31 while that of the ESP module is 17. After all branch convolutions are complete, features from all paths are concatenated in the channel dimension. Then, 1×1 convolution is adopted to prune feature channels. Finally, we add the input features to the intermediate features to obtain the output of the CDCM.

By using the ESP module as the basic building block, ESPNet [24] only contains 0.4 M parameters, but this also causes a low segmentation accuracy. Specifically, the MIoU of ESPNet on the Cityscapes test set reaches only 60%. For applications that require high precision, such as autonomous driving, this is far from satisfactory. Unlike ESPNet, we use the CDCM to replace the basic residual blocks in the last two stages of ResNet-18 [15], and achieve a better trade-off between model accuracy and efficiency by reasonably controlling the number of feature channels of the CDCM. Following the channel partition strategy in the Res2Net [41], given that input features have $C_{i}$ channels, the number of feature channels $C_{b}$ that need to be processed for each branch in the CDCM is calculated by Equation (3).

(3) $$C_{b}=C_{i}*\frac{width}{64}$$

Here, width is a hyper-parameter that controls the dimensionality of the feature maps. More specifically, when the number of input feature channels of CDCM is 256, we set the width to 24; thus, the number of feature channels for each branch is 96.

### 3.3. More Lightweight ResNet-18

Two backbone networks that are widely used to design real-time semantic segmentation networks are ResNet-18 [15] and MobileNetV2 [42]. The real-time semantic segmentation network based on Resnet-18 is generally more accurate than that based on MobileNetV2 due to the large number of parameters [26,27]. However the number of parameters of ResNet-18 is about three times that of MobileNetV2. We modify Resnet-18 to reduce its number of parameters and computations. By calculating the parameters of the convolution in each stage of ResNet-18, we find that the convolution in the last stage contains over 75% of the parameters of the whole network (after removing the full connection layers). The main reason for this is that the number of feature channels processed by these convolutions reaches 512. Note that ResNet-18 is mainly employed for object classification, and each layer of ResNet-18 extracts single-scale features under a fixed receptive field. Apart from the full connection layers, ResNet-18 only contains eight residual blocks, an initial convolutional layer, and a maximum pooling layer. Such a shallow network limits the receptive field. For all of the above reasons, we replaced the residual blocks in the last two stages of ResNet-18 with the CDCM to effectively increase the size of receptive fields and generate multi-scale feature maps. We further adjusted the number of feature channels in the last stage of ResNet-18 to 256 for efficient computation and a reduced number of parameters. The modified ResNet-18 is called multi-scale ResNet-18 (MSResNet-18) in this paper. MSResNet-18 has fewer parameters and computation costs than ResNet-18. See Section 4.2.1 for a detailed comparison.

### 3.4. Spatial Detail Module (SDM)

**Figure 6:**
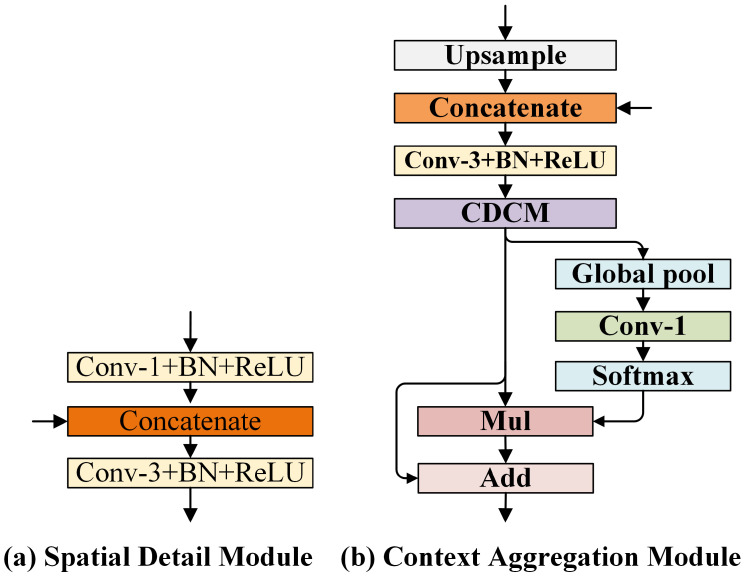
Structures of spatial detail module and context aggregation module. The “Conv-i+BN+ ReLU” block indicates the i×i convolution followed by batch normalization and ReLU. The “upsample” block indicates bilinear upsampling. The “Mul” block and "Add” block denote element-wise multiplication and element-wise summation, respectively. The "CDCM” block is the proposed cascade dilated convolution module.

The purpose of the SDM is to obtain as much spatial detail information as possible similar to that obtained by some useful traditional methods, such as dilated convolution [5], multi-branch sub-network [27], and encode–decode structure [12]. The excellent performance of these methods on many benchmark datasets proves the importance of spatial detail information for semantic segmentation. The high-resolution feature maps obtained by these methods not only require more computation resources, but also slow down computation, as mentioned in the introduction. In order to ensure computational efficiency, our SDM is designed as lightweight as possible. The structure of the SDM is shown in Figure 6a. Note that, when the SDM only has one input, the “concatenate” block in Figure 6a is removed.

Extensive ablation studies have demonstrated that adopting the output from Stage 2 to Stage 3 delivers a better trade-off in terms of overall performance compared with other options. Therefore, the proposed SDM has two inputs: $F_{stage_{2}}=\left\{f_{l_{1}}^{H\times W},f_{l_{2}}^{H\times W},\cdots ,f_{l_{C_{1}}}^{H\times W}\right\},$$F_{stage_{3}}=\left\{f_{l_{1}}^{H\times W},f_{l_{2}}^{H\times W},\cdots ,f_{l_{C_{2}}}^{H\times W}\right\},\left(C_{2}=64,C_{3}=128\right)$. First, in order to reduce the amount of computation of the SDM, a 1×1 convolution (stride = 2) is used to downsample the high-resolution feature map $F_{stage_{2}}^{}$ to achieve the same resolution as the low-resolution feature $F_{stage_{3}}$:

(4) $$F_{stage_{2}}^{{}^{\prime }H\times W\times C_{o}}=\delta \left(W^{1\times 1}\times F_{stage_{2}}^{H\times W\times C_{i}}+b\right),$$

where $W^{1\times 1}$ is defined as a 1×1 convolution, *b* represents the bias vector, and δ(·) indicates the operations of batch normalization (BN) [43] and the rectified linear unit (ReLU) [44] function. Subsequently, the downsampled features $F_{stage2}^{{}^{\prime }}$ and low-resolution features $F_{stage3}$ are concatenated in the channel dimension. Finally, a 3×3 convolution is used to blend the concatenated features, and the features with sufficient spatial detail information are obtained. We present this process, as follows:

(5) $$F_{SDM}^{H\times W\times C_{o}}=\delta \left(W^{3\times 3}\times C\left[F_{stage_{2}}^{{}^{\prime }H\times W\times C_{i}},F_{stage_{3}}^{H\times W\times C_{i}}\right]+b\right),$$

where $W^{3\times 3}$ is defined as a 3×3 convolution, and C[⋯] is the channel concatenation. Because only two convolutions are used, the time consumption is only slightly increased. At the same time, the low-level feature’s channel capacity is low and, thus, it will not bring too much memory consumption.

### 3.5. Context Aggregation Module (CAM)

After the SDM obtains enough spatial detail information, the purpose of the CAM is to gather multi-scale context information and channel-wise context information. The existing multi-scale contextual methods (based on spatial pyramid pooling) concentrate on spatial context information and usually ignore channel-wise context information [6,8]. As a matter of fact, each channel of a feature map often has a different importance. Some channels encode more spatial and context information than other channels [45]. One widely used technique is to adopt a global pooling operation that can encode the importance of output features into an attention vector. The different feature channels are weighted according to the attention vector (also known as channel attention). In BiSeNet [27] and SENet [46], global pooling is used to generate an attention vector for guiding feature learning, which enhances feature representation.

We combine the advantages of the above two architectures (spatial context and channel context) to extract multi-scale spatial context information and channel-wise context information simultaneously. As depicted in Figure 6b, given high-level semantic feature maps (that are generated by stage 4 and stage 5, respectively): $F_{stage_{4}}=\left\{f_{l_{1}}^{H\times W},f_{l_{2}}^{H\times W},\cdots ,f_{l_{C_{4}}}^{H\times W}\right\}$ and $F_{stage_{5}}=\left\{f_{l_{1}}^{H\times W},f_{l_{2}}^{H\times W},\cdots ,f_{l_{C_{5}}}^{H\times W}\right\}.\left(C_{4}=C_{5}=256\right)$ as an input of the CAM. We first upsample $F_{stage5}$ to the same resolution as $F_{stage4}$, and we then concatenate $F_{stage4}$ and $F_{stage5}$ in the channel dimension. Next, the feature map is blended by a 3×3 convolution, and it passes through the batch normalization layer and ReLU layer, as formulated in Equation (6).

(6) $$F_{stage_{4}}^{{}^{\prime }H\times W\times C_{o}}=\delta \left(W^{3\times 3}\times C\left[U\left(F_{stage_{5}}^{H\times W\times C_{i}}\right),F_{stage_{4}}^{H\times W\times C_{i}}\right]+b\right)$$

Here, U(·) represents the bilinear interpolation operation. A 3×3 convolution is used to reduce the number of feature channels (from 512 to 256) in order to reduce the amount of computations of the CDCM. In order to obtain multi-scale spatial contextual information, $F_{stage4}^{{}^{\prime }}$ are successively sent to the CDCM, and the dilated rates of the CDCM are set as D={1,5,9,17} to maintain the field of view. The output of the CDCM is defined as $F_{CDCM}$. The CDCM enables all pixels in the feature map to distinguish which adjacent points are more important. However, they cannot distinguish which feature channel encoding information is more important. To tackle this dilemma, we reshape the multi-scale feature $F_{CDCM}$ into feature vectors $V=\{v_{1}^{1\times 1},v_{2}^{1\times 1}\cdots ,v_{C_{i}}^{1\times 1}\}$ ($C_{i}$ is set as 256) via a global average pooling layer. Subsequently, we feed *V* into a 1×1 convolution and a softmax layer, similar to what is done in SENet [46]. After that, the attention vector that is generated by global pooling is multiplied with $F_{CDCM}$. Moreover, when considering its merits, we take the shortcut that is presented in [15] to reuse the feature maps $F_{CDCM}$ and facilitate information flow. We present this process, as follows:

(7) $$v_{k}=\frac{1}{H\times W}\sum_{i=1}^{H}\sum_{j=1}^{W}f_{CDCM}\left(i,j\right),$$

(8) $$f_{{cam}_{k}}^{H\times W}=\frac{\exp\left(v_{k}\right)}{\sum_{k=1}^{C_{o}}\exp\left(v_{k}\right)}⊕f_{{CDCM}_{k}}^{H\times W}⊗f_{{CDCM}_{k}}^{H\times W},$$

where $f_{{cam}_{k}}^{W\times H}$ refers to the k-th weighted output feature map, and $k\in \{1,2,\cdots ,C_{o}\}$, ($C_{o}$ is set as 256) ⊕ and ⊗ refer to element-wise multiplication and element-wise summation, respectively.

### 3.6. Decoder

**Figure 7:**
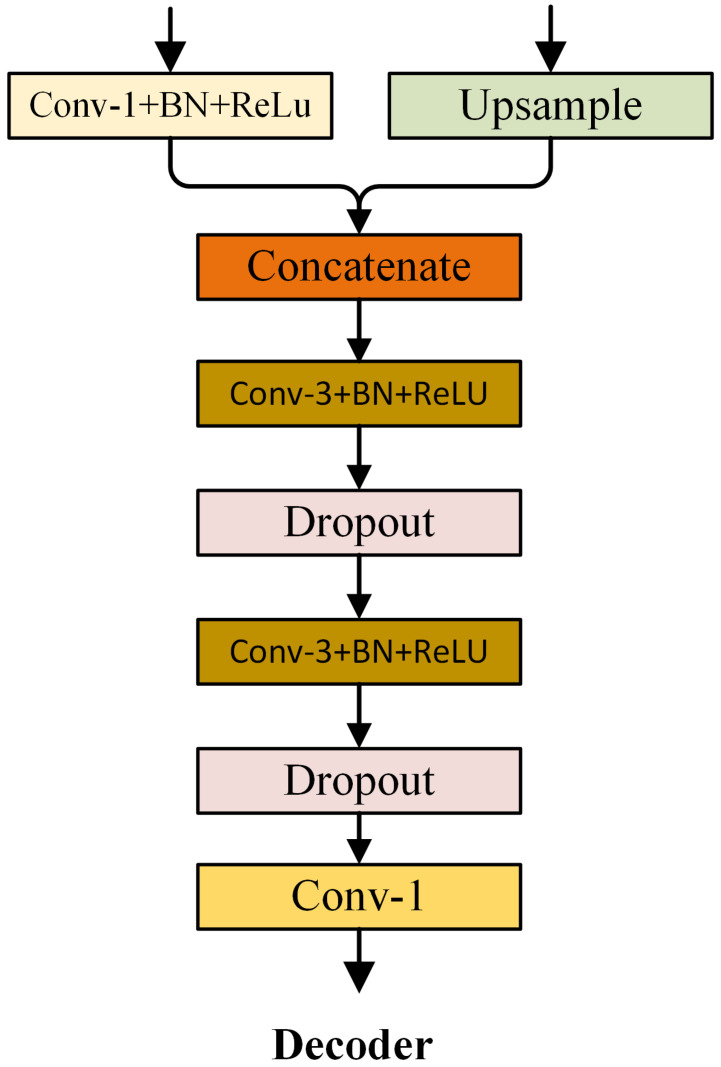
Details of the Decoder. The "dropout” block indicates the dropout layer.

Feature maps with different representation levels are obtained after the low-level and high-level features of the backbone network are processed by the SDM and CAM, respectively. We use the decoder structure in DeepLabV3+ [32] to obtain the final segmentation result. To speed up the calculation and reduce computational overhead, we reduce the number of feature channels that enter the decoder. Specifically, the number of channels of high-level semantic features is adjusted to 256, and the number of channels of low-level spatial detail features is adjusted to 32. Figure 7 shows the structure of the decoder.

## 4. Experiments

We use a modified ResNet-18 [15] model MSResNet-18 for real-time semantic segmentation tasks. We evaluate the proposed MMFANet on road driving datasets Cistyscapes [47] and CamVid [48]. First, the dataset and implementation protocol are introduced. Next, we performed ablation experiments for each part of the proposed method. All of the ablation experiments are carried out on the Cityscapes validation set. Finally, we compare the accuracy, speed, and model complexity of the proposed method with other state-of-the-art semantic segmentation methods on the above two datasets.

### 4.1. Datasets and Implementation Protocol

Cityscapes: Cityscapes is a collection of high-resolution images that were taken from a driver’s perspective on a clear day. It consists of a training set of 1975 images, a validation set of 500 images, and a testing set of 1525 images, all of which have a resolution of 2048 × 1024. These images were taken from 50 cities, with the shooting time distributed across seasons. The dataset is divided into 30 semantic categories. Following previous works [11,49], we use only 19 common semantic categories for training and evaluation in our experiments. More than 20,000 images with coarse annotations are also provided. In our experiments, we only use images with fine annotations.

CamVid: CamVid is also an urban street scene dataset related to autonomous vehicles. It contains 701 images, with 367, 101, and 233 images for training, validation, and testing, respectively. These images have a resolution of 960 × 720, and they belong to 32 classes. We refer to the index used in [5] to change the number of classes to 11. Following prior works [11,49], we randomly sub-sample all images to a 360 × 480 resolution to evaluate model performance.

Implementation details: our implementation is based on a public platform Pytorch (version 1.7.0). We use 5 GTX1080Ti cards under CUDA 9.0 and cuDNN V7 to train our model. For the loss function, the output resolution of our network is 1/8 of the input image and, thus, we upsample the final output with the same size as the input. Note that the different datasets use different loss functions. The standard cross-entropy loss is used for the CamVid dataset; for the Cityscapes dataset, we use the online hard example mining (OHEM) loss function because the Cityscapes scenes are more complex. The objective function is optimized using small batch stochastic gradient descent (SGD), the batch size is 10, the momentum decay is 0.9, and the weight decay is 0.025.

Learning rate strategy: similar to [5], we adopt a poly learning rate strategy, where the initial learning rate is multiplied by $lr=lr_{init}*{\left(\frac{1-iter}{max\_iter}\right)}^{power}$, and power = 0.9. Because the MSResNet-18 is not pre-trained on ImageNet [50], when experimenting on the Cityscapes and CamVid datasets, we set the maximum number of epochs to 1000 and 400, respectively.

Data augmentation: we use random horizontal flipping for all datasets, average subtraction, and random resizing between 0.75 and 2. Finally, images and labels in Cityscapes and CamVid are randomly cropped to 1024 × 1024 and 480 × 360 for training. Data augmentation was done using PyTorch’s data enhancement tool, which randomly enhanced the data during the training process. We did not generate any new data.

### 4.2. Ablation Study

In this subsection, we will study, in detail, the impact of each component on the proposed network. The following experiments are conducted with MSResNet-18 as the backbone when there are no special instructions. We evaluate our method on the Cityscapes validation dataset. We refer to the structure of FCN32s [34], and directly upsample the final output of the backbone network 32 times to the same size as the input image, and use the result of FCN32s as our baseline. Table 2 shows the experimental results.

#### 4.2.1. Ablation of the CDCM

The purpose of the CDCM is to extract multi-scale features while capturing a larger receptive field and improving the efficiency of ResNet-18, as mentioned in Section 3.2. It can be seen from Table 2 that, after replacing the residual blocks of the last two stages of ResNet-18 with the CDCM, the number of parameters is reduced by 8.5 M and the accuracy is improved from 62.31% to 64.88%. Furthermore, our MSResNet-18 achieves better accuracy than MobileNetV2, which adopts deep separable convolution to reduce the number of parameters.

To compare with the ESP [24] module, we construct a new model, called ESPResNet-18, where the CDCM in the MSResNet-18 is replaced by the ESP module. For fair comparison, the number of input feature channels of the ESP module is set as the same as that for the CDCM. We observe that ESPResNet-18 achieves comparable or slightly better performance in terms of both the number of parameters and FLOPs. However, the segmentation accuracy of ESPResNet-18 reduced to 61.56%, which is far from satisfying. The experimental results show the effectiveness and efficiency of the CDCM. After verifying the effectiveness of the CDCM, we set up another set of experiments to explore the dilated rate setting of the CDCM in MSResNet-18. We set up three different combinations of dilated ratios: {1,1,1,1},{1,2,4,8},{1,3,5,9} (named $D_{1}$, $D_{2}$, and $D_{3}$, respectively). Table 3 shows the experimental results. We can see that, by replacing standard convolution with a dilated convolution group D3, the performance improves from 62.72% to 64.88%. In the case of the same computational complexity and number of parameters, $D_{3}$ can achieve higher segmentation precision than $D_{2}$; this is in agreement with the conclusion presented in [40] because the hybrid dilated convolution (HDC) mode can alleviate the gridding artifact problem. Therefore, we choose $D_{3}$ as the final architecture of our network.

#### 4.2.2. Ablation of the CAM

The CAM combines the CDCM and channel-wise attention to gather spatial context and channel context information. We add the CAM to the baseline in order to analyze the impact of the CAM on network performance. At the same time, to compare with current multi-scale modules (i.e., the ASPP module and PPM), we also replace the CAM with these two modules and train the model under the same experimental conditions. The number of input feature channels of ASPP and PPM is 256, because the final feature map of MSResNet-18 only contains 256 channels. The experimental results are shown in Table 4. After adding the CAM to the baseline network, the performance improved from 64.88% to 77.37%. Hence, the CAM can obtain a stronger feature representation with only 0.9 M parameters and 1.4 GFLOPs. The comparison experiment with ASPP module shows that the CAM not only reduces the number of parameters by 1.5 M, but it also improves the MIoU by 3.43%. In addition, comparative experiments with the PPM show that, although the PPM achieves excellent performance on model efficiency, it sacrifices accuracy. These results indicate that, for our model, the combination of spatial context and channel attention in the CAM is a more accurate approach than the current multi-scale modules that only consider spatial context. We also note that the CAM has slightly more FLOPs than the ASPP module and PPM. When considering the trade-off between accuracy and complexity, the CAM remains a better choice.

#### 4.2.3. Ablation of the SDM

**Figure 8:**
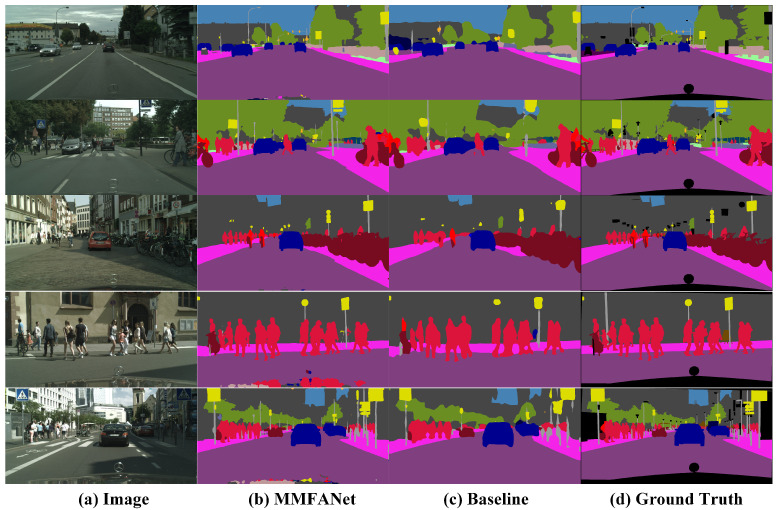
Examples of using MMFANet based on MSResNet-18 compared with the baseline on the Cityscapes validation dataset.

We select different feature maps as the input of the SDM to evaluate the quality of various low-frequency features combinations. All of the methods based on the SDM outperform the baseline method, as shown in Table 5. We observe that module c (the final architecture of the SDM) achieves comparable or slightly better performance in terms of both accuracy and efficiency than module a and module b. We attribute the superiority of module c to the fact that the output features of stage 2 and stage 3 of backbone network contain different scale information. The combination of these features is beneficial to the segmentation of various-scale objects in vehicle-mounted scenes. From the results presented in Table 5, we also find that the FLOPs of module a are about twice that of modules b, c, and d. The reason for this is that the resolution of module a’s feature map is 1/4 of the input image. Such a high-resolution feature map leads to intensive computation. In addition, the accuracy of module d, where a straightforward fusion of stage 2 and stage 3 features is implemented by channel concatenation, yields the least MIoU boost. This indicates that such fusion may improve the recognition performance by only a limited degree, because it may introduce noise. In contrast, our SDM achieves a 12.83% increase in MIoU at a small cost of adding 0.3 M parameters and 4.6 GFLOPs. The significant improvement in baseline performance is due to two main reasons. First, spatial detail information that is gathered by the SDM can help the network output more refined prediction. Figure 8 shows some segmentation results. Obviously, MMFANet can obtain the most refined segmentation results (such as boundaries and contours). Second, before the SDM is added to the baseline model, the feature map is directly upsampled 32 times to obtain the segmentation result. An excessive upsampling step size leads to rough prediction. We also notice that, after adding the SDM, the FLOPs of MMFANet increase to some extent because high-resolution features require more computational cost. However, when considering the trade-off between model accuracy and complexity, a slight increase in the amount of calculation is acceptable. After adding the SDM and CAM simultaneously, the upsampling step size is reduced from 16 to 8. This improves the performance from 77.71% to 79.16%, as shown in Table 4.

### 4.3. Comparison with State-Of-The-Art Methods

Table 6 reports the model accuracy and complexity of MMFANet based on ResNet-18 and MSResNet-18. As can be seen, MMFANet-1 achieves a 2.1% higher MIoU improvement than MMFANet-2 under the premise of reducing the parameters by 8 M. Furthermore, the FLOPs of MFFANet-1 are reduced by 11.2 G when the resolution is 1536 × 768. This proves that MSResNet-18 can obtain larger receptive fields and robust multi-scale features in a more efficient way, which is beneficial in improving the performance of semantic segmentation.

Previous lightweight methods, such as ENet [49], SegNet [11], ESPNet [24], DABNet [19], and LEDNet [20] achieve excellent performance on model efficiency. However, these methods sacrifice too much segmentation accuracy for efficiency. In contrast, the proposed MMFANet, which obtains 79.3% MIoU and yields a real-time speed of 58.5 FPS on a 1536 × 768 resolution image, makes an obvious improvement in both accuracy and efficiency. At the same time, when compared with recent state-of-the-art real-time semantic segmentation networks that are based on ResNet-18 (such as BiSeNet2 [27] and SwiftNet [26]), MMFANet also achieves higher accuracy with fewer parameters (the number of parameters is reduced by at least 53.38%). Our model also achieves better performance than some early non-real-time semantic segmentation networks (based on large-scale backbone), such as DeepLabV2 [5] and FCN8s [34], as shown in the top rows of Table 6. These results indicate that the proposed model can make full use of the features of all levels in the backbone network.

#### 4.3.1. Speed Analysis

In practical applications, speed determines whether a semantic segmentation network can produce practical effects. It is difficult to make a fair comparison because different methods use different hardware and software environments to test speed. In this experiment, we use a single 1080Ti GPU card as hardware and PyTorch as the software environment. In this process, we do not use any testing techniques or any accelerated optimization technique, such as multi-scale inputs or TensorRT implementation. We report the speed of our network at different input resolutions. MMFANet-1 can process 1024 × 2048 images at a speed of 33.5 FPS, as shown in Table 7. Because dilated convolution is not efficiently optimized, when the image has a low resolution, the speed of MMFANet-1 is slower than that of MMFANet-2.

#### 4.3.2. Performance Analysis on an Edge Device

We run a speed test on a Samsung S20 phone to test the proposed network’s performance on edge devices. When compared with professional deep learning computing platforms, smart phones have some gaps in computing performance. To this end, we compare the computing power of the Samsung S20 phone and Nvidia TX2; the specific data are shown in Table 8. As can be observed, the performance of the Samsung S20 is worse than that of the professional Nvidia TX2 device. Table 9 compares the speed of some real-time semantic segmentation models with the presented approach. Our code runs under float32 and it does not have some optimizations, such as TensorRT. The test code is based on PyTorch-Mobile. It can be seen that the model that is proposed in this paper can achieve a speed of about 10.3 FPS when the image resolution is 224 × 224. Moreover, the proposed method is faster than some methods that are based on custom modules, such as DABNet [19], LEDNet [20], and ESPNet [24]. The main reason for this may be that these networks only subsample the features eight times in order to retain more spatial details, resulting in too large of a feature resolution.

From Table 9 ut can be seen that the MSResNet-18 (MMFANet-1) proposed in this paper can achieve faster speed on mobile devices than ResNet-18 (MMFANet-2). This is the opposite of what was found in the server GPU test (Table 6). The reason for this phenomenon is that server GPUs have greater parallelism and may not be as sensitive to feature channel capacity as mobile devices. At the same time, we can also see that the MMFANet-1 that is based on MSResNet-18 is faster than some real-time semantic segmentation networks based on ResNet-18, such as SwiftNet and BiSeNet. The above experimental results verify that our optimization method of direct channel reduction can make the backbone network run faster on mobile devices.

### 4.4. CamVid

We conduct experiments on the CamVid dataset to verify the generalization of the proposed approach. Training and test images both have a 360 × 480 resolution. Table 10 shows the results for each class. It can be seen that the proposed method can achieve higher accuracy, even with lower-resolution images. Therefore, it can be inferred that our method has good generalizability.

## 5. Conclusions

MMFANet is proposed to deal with the multi-scale object problem of semantic segmentation in vehicle-mounted scenes, while striking a trade-off between model accuracy and efficiency. We propose three key modules: the CDCM, SDM, and CAM. The CDCM with different receptive field convolution branches is used to extract multi-scale features layer-by-layer and expand the receptive fields of the network. Furthermore, we use the CDCM to improve the efficiency of ResNet-18. The SDM only uses two convolutions and it is applied to extract spatial detail information, which improves the prediction details of the network without increasing the number of parameters too much. Meanwhile, the CAM combining the CDCM and channel attention is exploited to encode diverse contextual information and aggregate multi-scale features. The experiments show that the proposed MMFANet not only achieves precise segmentation results, but also reduces the model complexity. Without using any pretrained parameters, the whole network achieves 79.3% MIoU on the Cityscapes test set with 11.5 G FLOPs and a speed of 58.5 FPS; it also obtains a 68.1% MIoU on the CamVid test set.

## Figures and Tables

**Table 1:** Basic notations for the proposed method.

Notations	Meaning
H×W	The size of feature maps
$C_{i}$	The number of input channels
$C_{o}$	The number of output channels
$F_{{stage}_{i}}$	The output feature maps of the i-th stage in backbone network
$F_{{stage}_{i}}^{{}^{\prime }}$	The feature maps with same resolutions as $F_{{stage}_{i}}$
*D*	The set of dilation rates in CDCM
*x*	The input feature map of i-th branch of CDCM
$y_{i}$	The output of the i-th branch of CDCM
$F_{CDCM}$	The output of CDCM
*V*	The set of attention vectors for $F_{CDCM}$
$f_{l_{C_{i}}}^{H\times W}$	The i-th single channel feature map with resolution H×W

**Table 2:** Accuracy and model complexity analysis of MSResNet-18, ResNet-18, and MobileNetV2 on cityscapes validation dataset. For ESPResNet-18, we replace the CDCM in MSResNet-18 with the ESP module. Here, we use FCN-32s as the base structure. The number of float-point operations (FLOPs) are estimated for an input of 3×640×360.

Model	FLOPs (G)	Number of Parameters (M)	MIoU (%)
MSResNet-18	6.4	3.9	64.88
ESPResNet-18	5.1	2.0	61.56
ResNet-18	8.8	12.4	62.31
MobileNetV2	1.5	2.6	61.42

**Table 3:** A comparison of the CDCM under different dilated rates. In MSResNet-18-1, the dilated rates are {1,1,1,1}. In MSResNet-18-2, the dilated rates are {1,2,4,8}. In MSResNet-18-3, the dilated rates are {1,3,5,9}.

Approach	MIoU (%)	Number of Parameters (M)
MSResNet-18-1	62.72	3.9
MSResNet-18-2	63.37	3.9
MSResNet-18-3	64.88	3.9

**Table 4:** Detailed performance comparison of each component in our proposed MMFANet. CAM: context aggregation module; SDM: spatial detail module; ASPP: atrous spatial pyramid pooling; PPM: pyramid pooling module. Note that FLOPs are estimated on a 3×640×360 input.

Approach	MIoU (%)	Number of Parameters (M)	FLOPs (G)
Baseline (base on MSResNet-18)	64.88	3.9	6.4
Baseline + CAM	77.37	4.8	7.8
Baseline + PPM	74.41	4.7	6.5
Baseline + ASPP	73.94	6.3	6.8
Baseline + CAM + SDM	79.16	5.5	12.0

**Table 5:** Performance comparisons with various spatial detail modules. $S_{i}$: The output feature maps of the i-th stage of the backbone network. concatenate: Use “concatenate” to fuse feature maps. SF is the straightforward fusion of stage 2 and stage 3 features implemented by channel concatenation. Note that FLOPs are estimated on a 3×640×360 input.

Approach	$S_{2}$	$S_{3}$	Concatenate	SF	MIoU (%)	Number of Parameters (M)	FLOPs (G)
Baseline					64.88	3.9	6.4
a	✓				76.87	4.2	24.67
b		✓			76.28	4.2	10.54
c	✓	✓	✓		77.71	4.2	11.00
d	✓	✓		✓	75.19	4.15	10.73

**Table 6:** Accuracy and model complexity comparison of our method with state-of-the-art methods on the Cityscapes dataset. “-” indicates that the result is not provided. MIoUt and MIoUv denote the accuracy obtained on the Cityscapes test and validation sets, respectively. MMFANet-1 and MMFANet-2 are versions of MMFANet based on MSResNet-18 and ResNet-18, respectively.

Model	Resolution	Time (ms)	FPS	MIoUt (%)	MIoUv (%)	Number of Parameters (M)	FLOPs (G)
DeepLabV2 [5]	1024 × 512	-	0.3	70.4	71.4	44.0	457.8
FCN8s [34]	1024 × 512	-	2.0	65.3	-	134.5	136.2
PSPNet [8]	713 × 713	1288	0.78	80.2	-	250.8	412.2
DeepLabV3+ [32]	-	-	-	82.1	79.14	-	-
SegNet [11]	640 × 360	60	16.7	57	-	29.5	286.0
DABNet [19]	1024 × 2048	36	27.7	70.1	69.1	0.76	-
LEDNet [20]	-	14	71	70.6	-	0.94	-
ENet [49]	640 × 360	7	135.4	58.3	-	0.4	3.8
ICNet [30]	1024 × 2048	33	30.3	69.5	67.7	26.5	28.3
ERFNet [23]	1024 × 512	24	41.7	69.7	71.5	2.1	27.7
ESPNet [24]	1024 × 512	9	112	60.3	-	0.4	-
BiSeNet1 [27]	640 × 340	5	203.5	68.4	69.0	5.8	2.9
BiSeNet2 [27]	640 × 340	8	129.4	74.7	74.8	49.0	10.8
DFANetA [19]	1024 × 1024	10	100	71.3	71.9	7.8	3.4
DFANetB [19]	1024 × 1024	8	120	67.1	68.4	4.8	2.1
SwiftNetRN-18 [26]	1024 × 2048	-	39.9	75.5	75.4	11.8	104.0
MMFANet-1	1536 × 768	17.0	58.5	79.3	79.2	5.5	61.03
MMFANet-2	1536 × 768	16.5	60.71	77.2	78.5	13.5	72.23

**Table 7:** The speed of our method at different image resolutions. Image size is W × H. MMFANet-1 and MMFANet-2 are versions of MMFANet based on MSResNet-18 and ResNet-18 models, respectively.

	NVIDIA GTX1080-Ti						
**Model**	640×360		1280×720		1024×2048		**MIoU (%)**
	**ms**	**fps**	**ms**	**fps**	**ms**	**fps**	
MMFANet-1	12.75	78.41	14.87	67.24	29.85	33.50	76.9
MMFANet-2	7.96	125.61	14.63	68.34	28.66	34.89	76.1

**Table 8:** Comparison of performance parameters between Samsung S20 and Nvidia TX2.

Device	FLOPs	CPU	Memory
Nvidia TX2	1.33 TFLOPs	dual core NVIDIA Denver 2 64 bit CPU and quad Arm Cortex-A57 MPCore	128-bit LPDDR4 59.7 GB/s
Samsung S20	1.25 TFLOPs	64-bit dual ARM Cortex-A72 CPU and quad Cortex-A53 CPU	LPDDR4X Quad-channel (64-bit) 2133 MHz (34.1 GB/s)

**Table 9:** Speed of the proposed method as compared with some real-time semantic segmentation methods on mobile devices. MSResNet-18Fcn is our baseline network.

Approach	Image Resolution	FPS
MMFANet-1	224 × 224	10.3
MMFANet-2	224 × 224	6.8
DABNet [19]	224 × 224	8.0
ESPNet [24]	224 × 224	7.6
LEDNet [20]	224 × 224	6.1
BiSeNet(ResNet-18) [27]	224 × 224	7.2
SwiftNet(ResNet18-single-scale) [26]	224 × 224	6.1
MSResNet-18Fcn	224 × 224	11.8

**Table 10:** Accuracy results on the CamVid test dataset. MMFANet is based on MSResNet-18

Model	Building	Tree	Sky	Car	Sign	Road	Pedestrain	Fence	Pole	Sidewalk	Bycycle	MIoU (%)
SegNet [11]	88.8	87.3	92.4	82.1	20.5	97.2	57.1	49.3	27.5	84.4	30.7	55.6
ENet [49]	74.7	77.8	95.1	82.4	51.0	95.1	67.2	51.7	35.4	86.7	34.1	51.3
BiSeNet1 [27]	82.2	74.4	91.9	80.8	42.8	93.3	53.8	49.7	25.4	77.3	50.0	65.6
BiSeNet2 [27]	83.0	75.8	92.0	83.7	46.5	94.6	58.8	53.6	31.9	81.4	54.0	68.7
DABNet [19]	80.4	73.9	91.1	83.0	44.0	94.2	56.2	38.8	30.1	79.5	58.2	66.3
MMFANet	83.0	76.5	91.2	86.8	46.7	94.2	58.8	42.2	30.0	80.7	59.5	68.1

## Data Availability

No new data were created or analyzed in this study. Data sharing is not applicable to this article.
